# Transcorrelated Theory with Pseudopotentials

**DOI:** 10.1021/acs.jctc.5c00343

**Published:** 2025-05-13

**Authors:** Kristoffer Simula, Evelin Martine Corvid Christlmaier, Maria-Andreea Filip, J. Philip Haupt, Daniel Kats, Pablo Lopez-Rios, Ali Alavi

**Affiliations:** † Max Planck Institute for Solid State Research, Heisenbergstr. 1, 70569 Stuttgart, Germany; ‡ Yusuf Hamied Department of Chemistry, 28326 University of Cambridge, Lensfield Road, Cambridge CB2 1EW, United Kingdom

## Abstract

The transcorrelated (TC) method performs a similarity transformation on the electronic Schrödinger equation via Jastrow factorization of the wave function. This has demonstrated significant advancements in computational electronic structure theory by improving basis set convergence and compactifying the description of the wave function. In this work, we introduce a new approach that incorporates pseudopotentials (PPs) into the TC framework, significantly accelerating Jastrow factor optimization and reducing computational costs. Our results for ionization potentials, atomization energies, and dissociation curves of first-row atoms and molecules show that PPs provide chemically accurate descriptions across a range of systems and give guidelines for future theory and applications. The new pseudopotential-based TC method opens possibilities for applying TC to more complex and larger systems, such as transition metals and solid-state systems.

## Graphical abstract

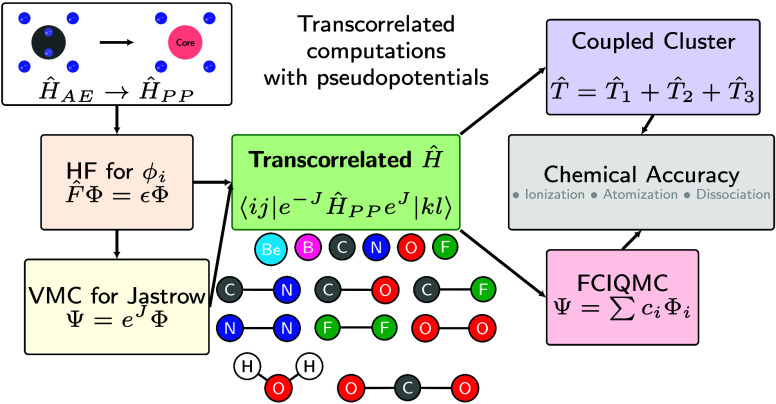

## Introduction

In methods based on electronic structure theory, solutions to the Schrödinger equation require simultaneous convergence with respect to basis sets and the treatment of electron correlation. Current ab initio methods that treat the electron correlation explicitly can achieve this accurately only for very small systems, due to the high polynomial or even exponential scaling of the computational cost with the number of electrons.

First quantized methods, such as Variational and Diffusion Monte Carlo (VMC and DMC), work in continuum space, circumventing the basis set issue. However, their accuracy is limited by the quality of the trial wave function. Systematic improvement of the wave function requires methods capable of treating electron correlation effectively.

Conversely, methods such as Coupled Cluster (CC) and configuration interaction (CI), formulated under the framework of second quantization, can often treat electron correlation accurately, but convergence to the basis set limit remains infeasible in many cases. Additionally, the divergent nature of the Coulomb interaction introduces sharp features, or cusps,^1^ in the wave function, which are difficult to describe using determinants composed of smooth Gaussian-type orbitals, which are often used in second-quantized post-Hartree–Fock methods.

These issues of second quantization have been alleviated by explicitly correlated methods, which introduce interelectronic distance dependency in the system description. This is done to reduce basis set errors and account for the cusps, and to describe short-range electronic interactions without requiring a large number of determinants.

R12/F12 methods^2,3^ have been widely applied in quantum chemistry to address these problems, and have been used in perturbation theory, coupled cluster, and configuration interaction calculations,^4–6^ generally with good results.

The transcorrelated (TC) method is an explicitly correlated method that has seen rapid development in recent years.^7–12^ TC takes a Jastrow factor, a function of interelectronic distances optimized in a first-quantized VMC calculation, and uses it to perform a similarity transformation on the second-quantized Hamiltonian. This transformation preserves the Hamiltonian’s eigenvalues while addressing the cusps and significantly improving basis set convergence,^13^ as well as compactifying the wave function.^12–14^ Although the transformation introduces challenging three-body terms, a recent approximation, xTC,^15^ removes the need to explicitly treat these terms, reducing the scaling in evaluation of the transcorrelated Hamiltonian by 2 orders of magnitude.

TC has shown very promising results in homogeneous electron gas (HEG) systems,^16,17^ and the Hubbard model.^14^ It has also been applied to atoms and molecules with high accuracy using FCIQMC and CC methods.^7,18–20^ TC has also been used to produce accurate results in simulations with quantum hardware, where the TC Hamiltonian enables the use of shallower circuit depths.^21^

One of the bottlenecks of TC and xTC has been the optimization of the Jastrow factor, as the variance of the VMC wave function increases rapidly with system size, driving up computational costs for sufficient accuracy. To extend TC and xTC to larger systems or solids, further developments are needed to reduce the costs of Jastrow optimization and the following post-Hartree–Fock calculations. The pseudopotential (PP) approximation offers a promising solution, as it reduces the number of electrons, eliminates electron–nucleus cusps, and significantly lowers the VMC variance.

In this work, we investigate the use of PPs in xTC methods. Replacement of the nucleus and the surrounding core electrons with PPs introduces terms in the Hamiltonian that do not commute with the Jastrow factor, complicating the similarity transformation. In the Theory section, we present the theory of transcorrelation with PPs. In the Calculations section, we discuss the computational details of our calculations. The Results section presents results on ionization potentials for first-row elements (Be–F), atomization energies for molecules (CN, CO, CF, N_2_, O_2_, F_2_, H_2_O, CO_2_), and dissociation curves for N_2_ and F_2_. We show that PPs accelerate the optimization of the Jastrow factor and provide chemically accurate descriptions across a variety of systems and chemical environments.

## Theory

### Pseudopotential Approximation

Within the PP approximation the Coulombic interactions between the valence electrons and the atomic nuclei and core electrons are replaced with effective potentials. The PPs are constructed to reproduce the valence electron wave functions outside of a core region. This means that the electron–nucleus term of the Hamiltonian *Ĥ*_en_ is replaced by a sum over effective potentials *V̂*_eff_ for each atom

1 $${Ĥ}_{\mathrm{en}}=\sum_{I}^{M}\sum_{i=1}^{N}\frac{Z_{I}}{\left|r_{i}-R_{I}\right|}\to {Ĥ}_{\mathrm{en}}^{\mathrm{PP}}=\sum_{I}^{M}\sum_{j=1}^{N_{v}}{\mathrm{V̂}}_{\mathrm{eff}}^{I}\left(|r_{j}-R_{I}|\right)$$

Above, *Z* is the nuclear charge, *N* (*M*) is the number of electrons (atoms), and **r**_*i*_ (**R**_*I*_) is the position of the *i*th electron (*I*th atom) with respect to the origin. With PPs, the summation runs over *N*_*v*_ valence electrons.

In the theory that follows, we focus on the effective potential of a single atom (*M* = 1) to simplify presentation, but the extension to multiatom systems is straightforward.

Outside of the core region *V*_eff_^*I*^ should mimic the potential felt by the valence electrons due to the nucleus and the core electrons. It should also reproduce the exact electronic wave function outside of the core region specified by a cutoff radius *r*_c_. *V*_eff_ consists of a number of angular momentum channels, one local without spherical projections and one or more nonlocal channels

2 $${\mathrm{V̂}}_{\mathrm{eff}}\left(r\right)=V_{l_{\max}}\left(r\right)+\sum_{l=0}^{l_{\max}-1}V_{l}\left(r\right)\sum_{m=-l}^{l}\left|Y_{\mathrm{lm}}\right\rangle \left\langle Y_{\mathrm{lm}}\right|$$

Above, *Y*_*lm*_ are the spherical harmonics, and *V*_*l*_(*r*) are the pseudopotential radial functions. *l*_max_ is the maximum angular momentum quantum number included in the PP, which is also chosen as the local channel. *V*_*l*_ are expressed as

$$V_{l}\left(r\right)=\{\begin{array}{cc} -\frac{Z_{\mathrm{eff}}}{r}\left(1-e^{-\alpha r^{2}}\right)+\alpha Z_{\mathrm{eff}}re^{-\beta r^{2}}+\overset{n}{\underset{q=1}{\sum }}\gamma_{\mathrm{ql}}e^{-\delta_{\mathrm{ql}}r^{2}} & l=l_{\max}\\ \overset{m}{\underset{q=1}{\sum }}\gamma_{\mathrm{ql}}e^{-\delta_{\mathrm{ql}}r^{2}} & l<l_{\max} \end{array}$$ 3

We use two sets of PPs in this work: energy-consistent correlated electron PPs (eCEPPs) by Trail and Needs,^22^ and correlation-consistent effective core potentials (ccECPs) by Mitas et al.^23^ The eCEPPs have *l*_max_ = 2 for the first-row atoms, with *n* = 4 and *m* = 6, while the ccECPs have *l*_max_ = 1 and *n* = *m* = 1, leaving only one Gaussian term for the nonlocal channels. The different components of ccECPs and eCEPPs for C, N, O, and F are shown in Figure 1. In the figure, it can be seen that the eCEPPs have much smaller potential absolute values than the ccECPs.

The action of *V̂*_eff_ on a function *f*(**r**) (with or without a Jastrow factor) of the position of electron *i* is

4 $${\mathrm{V̂}}_{\mathrm{eff}}\left(r_{i}\right)f\left(r_{i}\right)=\sum_{l=0}^{l_{\max}}V_{l}\left(r_{i}\right)\sum_{m=-l}^{l}Y_{\mathrm{lm}}\left(\Omega_{r_{i}}\right)\int_{\left|r\prime \right|=r_{i}}d\Omega_{r\prime }f\left(r^{\prime }\right)Y_{\mathrm{lm}}\left(\Omega_{r\prime }\right)$$

Because *Y*_*lm*_(*f* = 0, θ = 0) = 0 for *m* ≠ 0, we can simplify this expression—by choosing the *z*-axis to be along **r**_*i*_—to^24^

5 $${\mathrm{V̂}}_{\mathrm{eff}}\left(r_{i}\right)f\left(r_{i}\right)=\sum_{l=0}^{l_{\max}}V_{l}\left(r_{i}\right)Y_{l0}\left(\Omega_{r_{i}}\right)\int_{\left|r\prime \right|=r_{i}}d\Omega_{r\prime }f\left(r^{\prime }\right)Y_{l0}\left(\Omega_{r\prime }\right)$$

which is the expression we use to estimate the action of the PP on the electronic orbitals and the Jastrow factor.

### Pseudopotentials in the Transcorrelated Hamiltonian

In transcorrelation theory, the Hamiltonian is supplemented with a Jastrow factor *J*, which is a function to describe interparticle correlations. With *N* electrons and *M* nuclei in a system, the Jastrow factor in this work is of Drummond–Towler–Needs (DTN) type^25^

6 J({ri},{RI})=∑I=1M∑i=1Nχ(riI)+∑i<ju(|ri−rj|)+∑I=1M∑i<jf(riI,rjI,|ri−rj|)=∑I=1M∑i>jJ2(ri,rj;RI)

including 1-body (χ, electron–nucleus), 2-body (*u*, electron–electron), and 3-body (*f*, electron–electron–nucleus) terms. Each of the terms is optimized for interparticle distances under a chosen cutoff value, characteristic of the DTN Jastrow form. Above, **r**_*iI*_ = **r**_*i*_ – **R**_*I*_. In the above, in order to simplify the derivation of the pseudopotential commutator equations,^15^ we have included all of the terms involving χ, *u*, and *f* into the term *J*_2_(**r**_*i*_,**r**_*j*_,**R**_*I*_). This approach gives TC contributions to the 2- and 3-body terms ⟨*pr*|*qs*⟩ and ⟨*pqr*|*stu*⟩ of the Hamiltonian, including also the χ-term contribution to these terms. We call this approach the “combined” Jastrow treatment. Alternatively, one could treat the 1- and 2-body terms in the Jastrow factor separately (which we call the “separate” Jastrow treatment) and include the contribution of χ into the 1-body terms ⟨*p*|*q*⟩. The separate Jastrow treatment does not remove the contribution of the χ-term from the 2-body integrals, however, because of the presence of a cross-term (see Supporting Information). With small χ cutoff values the combined and separated treatments yield almost exactly the same coupled cluster total energies for atoms and molecules at equilibrium geometries, with discrepancy of <0.1 mHa. However, we found difficulties with the separated Jastrow treatment with PPs whenever the Jastrow cutoff values for the χ functions exceeded the internuclear bond lengths, and for this reason we chose the combined approach in this study. We present the equations for the separated treatment of the 1- and 2-body terms in the Jastrow factor in the Supporting Information.

The transcorrelated Hamiltonian is obtained from a similarity transformation of the original Hamiltonian

7 $${Ĥ}_{\mathrm{TC}}=\exp\left(-J\right)Ĥ\exp\left(J\right)=Ĥ+\left[Ĥ,J\right]+\frac{1}{2!}\left[\left[Ĥ,J\right],J\right]+...$$

Any operator component of the Hamiltonian Ô that does not commute with the Jastrow factor *J* will extend the transcorrelated Hamiltonian. For single-particle operators, the first two commutators can be expressed as

8 $$\begin{array}{l} \left[Ô,J\right]=\sum_{k=1}^{N}\sum_{i<j}\left[Ô\left(r_{k}\right),\sum_{I}^{M}J_{2}\left(r_{i},r_{j};R_{I}\right)\right]=\sum_{k=1}^{N}\sum_{i<j}\Pi_{\mathrm{ij}}^{k}\left(Ô\right)\\ \left[\left[Ô,J\right],J\right]=\sum_{k=1}^{N}\sum_{i<j}\sum_{l<m}\left[\left[Ô\left(r_{k}\right),\sum_{I}^{M}J_{2}\left(r_{i},r_{j};R_{I}\right)\right],\sum_{J}^{M}J_{2}\left(r_{l},r_{m};R_{J}\right)\right]=\sum_{k=1}^{N}\sum_{i<j}\sum_{l<m}\Gamma_{\mathrm{ijlm}}^{k}\left(Ô\right) \end{array}$$

It should be noted that the similarity transformation breaks the variational principle, although it does not affect the eigenvalues of the Hamiltonian.

**Figure 1:**
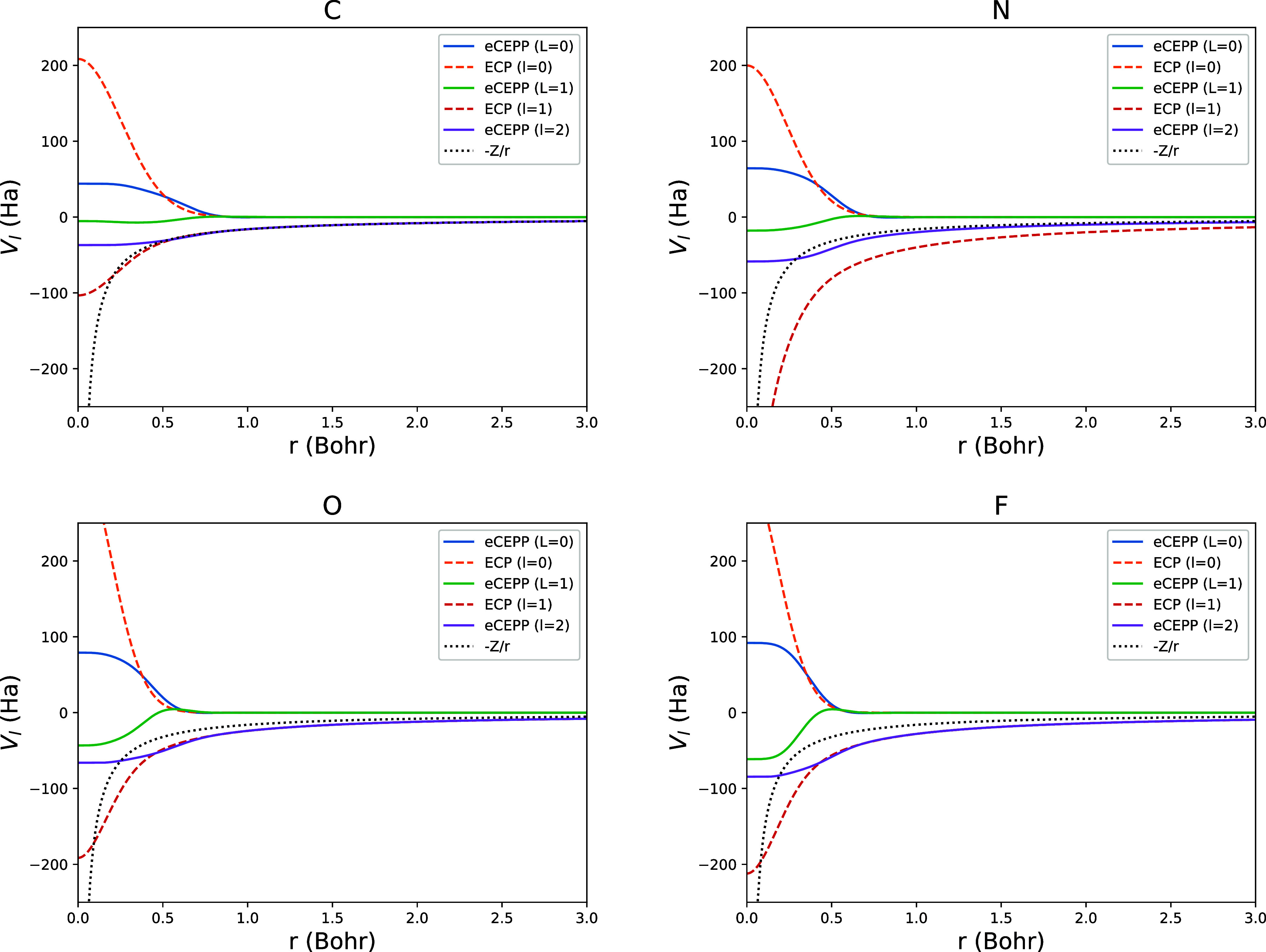
*V*_*l*_(*r*) of eCEPPs (solid lines) and ccECPs (dashed lines) of C, N, O, and F. Black dashed lines show the Coulombic potential of the nucleus.

There are restrictions to the indexing of Π and Γ terms. First, Π_*ij*_^*k*^ is nonzero only when *k* = *i* or *k* = *j*. Second, Γ_*ijlm*_^*k*^ is nonzero only when *k* = *i* and *i* ∈ (*l*, *m*) or *k* = *j* and *j* ∈ (*l*, *m*). Hence the commutators can be expressed as

9 $$\begin{array}{l} \left[Ô,J\right]=\sum_{i<j}\Pi_{\mathrm{ij}}^{i}\left(Ô\right)+\Pi_{\mathrm{ij}}^{j}\left(Ô\right)\\ \left[\left[Ô,J\right],J\right]=\sum_{i>j}\left[\Gamma_{\mathrm{ijij}}^{i}\left(Ô\right)+\Gamma_{\mathrm{ijij}}^{j}\left(Ô\right)\right]+\sum_{i>j>m}\left[\Gamma_{\mathrm{ijim}}^{i}\left(Ô\right)+\Gamma_{\mathrm{jijm}}^{j}\left(Ô\right)+\Gamma_{\mathrm{mimj}}^{m}\left(Ô\right)\right] \end{array}$$

In all-electron transcorrelation theory, the only component of the Hamiltonian that does not commute with the Jastrow factor is the kinetic energy operator.^7^ In this case the similarity transformation of eq 7 terminates exactly at the second commutator, and the transcorrelated Hamiltonian is computed with the commutators in eq 9, introducing 2- and 3-body terms in the Hamiltonian.

With PPs, the *V*_eff_ terms do not commute with the Jastrow factor. In addition, commutator summation in the similarity transformation is not guaranteed to terminate at the second commutator. To estimate the effect of the PP on the transcorrelated Hamiltonian, we make an approximation of only considering the 2-body terms of the PP commutators, ignoring the sum over *i* > *j* > *m* in eq 9. This approximation is made under the assumption that the 3-body interactions of the valence electrons close to the core are negligible. Explicit treatment of the 3-body terms, possibly under the xTC approximation, is left for future work in case it is needed. Within this approximation, the transcorrelated second-quantized Hamiltonian becomes

$$\begin{array}{l} Ĥ=\underset{\mathrm{pq}\sigma }{\sum }h_{q}^{p}a_{p\sigma }^{†}a_{q\sigma }+\frac{1}{2}\underset{\mathrm{pqrs}}{\sum }\left(V_{\mathrm{rs}}^{\mathrm{pq}}-K_{\mathrm{rs}}^{\mathrm{pq}}+P_{\mathrm{rs}}^{\mathrm{pq}}\right)\underset{\sigma \tau }{\sum }a_{p\sigma }^{†}a_{q\tau }^{†}a_{s\tau }a_{r\sigma }-\frac{1}{6}\underset{\mathrm{pqrstu}}{\sum }L_{\mathrm{uts}}^{\mathrm{pqr}}\underset{\sigma \tau \lambda }{\sum }a_{p\sigma }^{†}a_{q\sigma \prime }^{†}a_{r\lambda }^{†}a_{u\lambda }a_{t\tau }a_{s\sigma } \end{array}$$ 10

The terms *h* and *V* are traditional one- and two-body terms of the second-quantized Hamiltonian, while the calculation of the transcorrelated components *K* and *L* arising from the kinetic energy operator has been described elsewhere.^7,15^ The pseudopotential terms *P* are computed as

11 $$P_{\mathrm{rs}}^{\mathrm{pq}}=\left\langle \phi_{p}\phi_{q}\left|\Pi_{12}^{1}\left({Ĥ}_{\mathrm{en}}^{\mathrm{PP}}\right)+\Pi_{12}^{2}\left({Ĥ}_{\mathrm{en}}^{\mathrm{PP}}\right)+\frac{1}{2}\Gamma_{1212}^{1}\left({Ĥ}_{\mathrm{en}}^{\mathrm{PP}}\right)+\frac{1}{2}\Gamma_{1212}^{2}\left({Ĥ}_{\mathrm{en}}^{\mathrm{PP}}\right)\right|\phi_{r}\phi_{s}\right\rangle$$

with

12 $$\begin{array}{l} \Pi_{\mathrm{ij}}^{i}\left({Ĥ}_{\mathrm{en}}^{\mathrm{PP}}\right)=\sum_{I}^{M}\left[{Ĥ}_{\mathrm{en}}^{\mathrm{PP}}\left(r_{i}\right)J_{2}\left(r_{i},r_{j};R_{I}\right)-J_{2}\left(r_{i},r_{j};R_{I}\right){Ĥ}_{\mathrm{en}}^{\mathrm{PP}}\left(r_{i}\right)\right]\\ {\Gamma_{\mathrm{ijij}}^{i}\left({Ĥ}_{\mathrm{en}}^{\mathrm{PP}}\right)={Ĥ}_{\mathrm{en}}^{\mathrm{PP}}\left(r_{i}\right)\left[\sum_{I}^{M}J_{2}\left(r_{i},r_{j},R_{I}\right)\right]}^{2}-2\sum_{I}^{M}J_{2}\left(r_{i},r_{j};R_{I}\right){Ĥ}_{\mathrm{en}}^{\mathrm{PP}}\left(r_{i}\right)\sum_{J}^{M}J_{2}\left(r_{i},r_{j};R_{J}\right)+{\left[\sum_{I}^{M}J_{2}\left(r_{i},r_{j};R_{I}\right)\right]}^{2}{Ĥ}_{\mathrm{en}}^{\mathrm{PP}}\left(r_{i}\right) \end{array}$$

Therefore, in order to evaluate the PP commutators (under the present approximation of restricting ccECP corrections to two-body terms) in eq 11, we need to apply eq 5 to calculate terms of the type *Ĥ*_en_^PP^ϕ, *Ĥ*_en_^PP^ *J*_2_ϕ, and *Ĥ*_en_^PP^ *J*_2_^2^ϕ. This allows us to construct the transcorrelated Hamiltonian in eq 10. We have included also higher-order terms in the PP commutators in eq 12 in our calculations, under the assumption that the 3-body terms are negligible.

## Calculations

### Evaluation of Transcorrelated Hamiltonian with Pseudopotentials

The transcorrelated second-quantized Hamiltonians are calculated with an in-house code TCHINT,^26^ that is based on the version used in ref 7. TCHINT calculates transcorrelated second-quantized Hamiltonians with a Jastrow factor and the molecular orbitals as inputs. It is parallelized with MPI, uses the BLAS library for matrix operations, and is written in Fortran. The inclusion of the features necessary for the PP commutator evaluation within TCHINT has been an important part of this work.

The elements *P*_*rs*_^*pq*^ in eq 11 are obtained with numerical integration over real-space grid points. The operation of *Ĥ*_en_^PP^ in terms Π and Γ is evaluated according to eq 5, by discretizing the spherical integration, so that

13 $${\mathrm{V̂}}_{\mathrm{eff}}\left(r_{i}\right)f\left(r_{i}\right)=\sum_{l=0}^{l_{\max}}V_{l}\left(r_{i}\right)Y_{l0}\left(\Omega_{r_{i}}\right)\sum_{i=1}^{N_{s}}f\left({r_{i}}^{\prime }\right)Y_{l0}\left(\Omega_{r_{i}^{\prime }}\right)$$

where |**r**_*i*_′| = |**r**_*i*_| = *r*_*i*_ and *f* is either ϕ_*r*_(**r**_1_), *J*_2_(**r**_1_,**r**_2_) ϕ_*r*_(**r**_1_), or *J*_2_(**r**_1_,**r**_2_)^2^ϕ_*r*_(**r**_1_). The spherical grid points are chosen as the vertices of an icosahedron. The points are obtained by setting them on unit sphere scaled with *r*_*i*_, so that [±*a*, ±*b*, 0], [±*b*, ±*a*, 0], and [0, ±*a*, ±*b*]­are the grid points **r**_*i*_′ on the unit sphere, with $a=r_{i}/\sqrt{1+\phi^{2}}$ and $b=\phi r_{i}/\sqrt{1+\phi^{2}}$ , where $\phi =\left(1+\sqrt{5}\right)/2$ is the golden ratio. Hence *N*_s_ = 12. This is a Lebedev grid capable of exact spherical integration of functions that have up to *l* = 5 components. Tests on denser spherical grids did not change the results significantly. To mitigate the bias by the orientation of the spherical grid we applied random rotations to the spherical grid points in each spherical projection.

For a number of grid points *N*_ECP_ under pseudopotential influence, the numbers of additional Jastrow factor and orbital evaluations due to presence of pseudopotentials are *N*_g_ *N*_ECP_ *N*_s_ and *N*_ECP_ *N*_orb_ *N*_s_, respectively, where *N*_g_ is the total number of grid points and *N*_orb_ is the number of orbitals. The additional Jastrow evaluations thus add a *N*_ECP_ *N*_s_/*N*_g_ prefactor to the computational scaling of the Jastrow evaluations,^7^ which is computationally most expensive part of the calculation. To mitigate the additional computational cost and load imbalance, we use vectorized Jastrow evaluations and a load balancing scheme that distributes the grid points evenly among the MPI processes. Orbitals at the spherical grid points are evaluated at the beginning of the calculation, and the memory requirements are increased because of ECPs by *N*_ECP_ *N*_orb_ *N*_s_ additional floating point numbers as opposed to the *N*_g_ *N*_orb_ floating point numbers required for all-electron calculations.

Table 1 shows a list of the number of grid points under pseudopotential influence for a number of systems studied in this work. For each system, the number of pseudopotential-influenced grid points is roughly 55% of the total number of grid points.

**Table 1:** Total and ECP-Influenced Grid Points for Each System and Pseudopotential

system	total	eCEPP	ccECP
C	18,120	10,570	10,268
N	18,120	10,570	9966
O	18,120	10,268	9362
F	18,120	9966	9060
N_2_	36,196	22,792	20,810
O_2_	36,198	21,380	19,058
F_2_	36,202	20,316	18,324
CN	36,196	22,445	21,045
CO	36,196	22,022	20,313
CF	36,198	21,299	19,767
H_2_O	33,484	20,066	9612
CO_2_	54,056	33,384	30,250

The treatment of the 3-body terms when constructing the transcorrelated Hamiltonian is done under the xTC approximation.^15^ In this approximation, the last term of eq 10 containing *L* is reorganized within the generalized normal ordering scheme. This leads to modifications in the 1-, 2-, and 3-body terms of the transcorrelated Hamiltonian. The contributions of the 3-body terms under the generalized normal ordering to the 1- and 2-body terms are evaluated by contracting the 3-body terms with the reduced 1-body density matrix of the Hartree–Fock wave function with the exception of using the FCI density matrix in the N_2_ dissociation curve calculations. The remaining 3-body terms are neglected in xTC.

### General Computational Details

In this work we study the use of pseudopotentials with the transcorrelated Hamiltonian in atoms Be, B, C, N, O, and F, as well as their +1 ions. We also study the total and atomization energies of molecules CN, CO, CF, N_2_, O_2_, F_2_, H_2_O, and CO_2_. We use the aug-cc-pVD/T/QZ basis sets (AVXZ, with X = D, T, Q), optimized individually for each pseudopotential.^22,23^ Ionization energies with ccECPs are evaluated with nonaugmented cc-pVD/T/QZ basis sets (PVXZ, with X = D, T, Q).

The geometries of the molecules are taken from those in the HEAT database.^27^ The Hartree–Fock (HF) calculations are done with the PYSCF code.^28^ The Jastrow factors used are of Drummond–Towler–Needs type^25^ and are optimized with respect to the variance of the Hartree–Fock wave function using the VMC method of the CASINO package.^29^ The Jastrow factors are optimized separately for each system, basis set, and pseudopotential combination. The cutoffs used for the *u*, χ, and *f* terms were 4.5, 4, and 4, respectively. We provide the optimized Jastrow factors in the Supporting Information.

The atomization energies are calculated both with and without transcorrelation with the coupled cluster using singles, doubles and perturbative and full triples (CCSD­(T) and CCSDT) using the ElemCo.jl package.^30^ If transcorrelation is used, we add a prefix xTC- to the method name. The similarity transformation of the Hamiltonian using the Jastrow factor leads to a non-Hermitian Hamiltonian with a nondiagonal Fock matrix. Consequently, standard noniterative perturbative methods for CCSD­(T) are not directly applicable to the transcorrelated Hamiltonian. Thus, for transcorrelated CCSD­(T) we do the calculations with a pseudocanonical ΛCCSD­(T) approach using biorthogonal orbitals.^31^ To simplify notation we will call it xTC–CCSD­(T).

The atomization energies are also calculated with CCSD­(T)-F12 using the Molpro package^32–34^ for comparison. Both all-electron and PP F12 results are calculated to assess the effect of the PPs on the results. In all-electron calculations we use the standard AVXZ family of basis sets with AVXZ-MP2Fit and VXZ-JKFit auxiliary basis sets.^35^ In PP calculations we use the augmented basis sets fitted for PPs.^22,23^ As the auxiliary basis sets with PPs we use MP2-fitted QZVPP/MP2Fit and TZVPP/MP2Fit basis sets.

For the dissociation curves, we use the full configuration interaction quantum Monte Carlo method (FCIQMC)^36^ with the NECI package^37^ both with and without transcorrelated Hamiltonians. The initiator approximation^36^ with an initiator threshold of 3 is used in the FCIQMC calculations. The walker number for each calculation was increased by a factor of 5 until the energy was converged to within 1 mHa. We compare the results with MRCI-F12 calculations, done with the Molpro package.^32–34^ The Davidson correction^38^ is used in the MRCI-F12 calculations.

### Notation

All of the calculations presented in the following sections—with the exception of some of the F12-calculations—are done with PPs. To estimate the effect of evaluating the PP commutators we do the transcorrelated calculations without the PP commutators, and with varying level of commutator evaluations. We refer to these calculations as xTC-{method}­(PP-*n*), with *n* indicating the level of commutator evaluation (0–4 commutator evaluations in this work) and method indicating the method used (CCSD­(T), CCSDT, FCIQMC).

When presenting the total energies of atoms, ions, and molecules with PPs, we show the results relative to an energy *E*_CBS_^TZ–QZ^, evaluated as the sum of Hartree–Fock energy in the PVQZ (ccECPs) or AVQZ (eCEPPs) basis set, *E*_HF_^QZ^, and the estimate of the complete basis set limit (CBS) of CCSD­(T) correlation energy. This estimate is obtained from the PVTZ and PVQZ (ECP) or AVTZ and AVQZ (eCEPP) correlation energies *E*_TZ_^Corr^(CCSD­(T)) and *E*_QZ_^Corr^(CCSD­(T)) with a linear extrapolation, so that

14 $$E_{\mathrm{CBS}}^{\mathrm{TZ-QZ}}\left(\mathrm{CCSD}\left(T\right)\right)=E_{\mathrm{HF}}^{\mathrm{QZ}}+\frac{3^{3}E_{\mathrm{TZ}}^{\mathrm{Corr}}\left(\mathrm{CCSD}\left(T\right)\right)-4^{3}E_{\mathrm{QZ}}^{\mathrm{Corr}}\left((\mathrm{CCSD}\left(T\right))\right)}{3^{3}-4^{3}}$$

This estimation of the complete basis set limit energy with respect to the triple- and quadruple-ζ basis sets is not meant to serve as a benchmark, but rather as a reference point for the PP energies for easier comparison.

## Results

### Variances in VMC Optimization

Table 2 shows the variance of the reference energy in Hartrees for the first row elements Be–F, and for a set of first row molecules, obtained from eCEPP, ccECP, and all-electron (AE) calculations. For the results, we sampled the reference Hartree–Fock wave function with the Metropolis algorithm and evaluated an estimate of the variance of the obtained configurations together with the optimized Jastrow factor. The percentages after the PP variances show the ratio of PP values against the all-electron variances. The variance is significantly reduced when using PPs as compared to AE calculations. The variance reduction is greater for the heavier atoms. For the atoms, the variance reduction seems to obey roughly 1/*N*_*v*_ dependence, where *N*_*v*_ is the number of valence electrons.

**Table 2:** Variance Data for Atoms and Molecules Using AE, eCEPP, and ccECP Methods^a^

atom/molecule	AE	eCEPP	ccECP
Be	0.0586	0.0146 (25%)	0.0180 (31%)
B	0.224	0.045 (20%)	0.049 (22%)
C	0.510	0.085 (17%)	0.082 (16%)
N	1.110	0.144 (13%)	0.135 (12%)
O	2.290	0.254 (11%)	0.243 (11%)
F	4.300	0.400 (9%)	0.364 (8%)
N_2_	2.067	0.4441 (21%)	0.4313 (21%)
O_2_	3.734	0.7133 (19%)	0.6741 (18%)
F_2_	6.428	0.9423 (15%)	0.8828 (14%)
CN	1.482	0.3353 (23%)	0.3188 (22%)
CF	3.753	0.5674 (15%)	0.5336 (14%)
CO	2.483	0.4602 (19%)	0.4321 (17%)
H_2_O	1.763	0.3100 (18%)	0.2917 (17%)
CO_2_	4.236	0.7683 (18%)	0.7212 (17%)

a Units are in Hartrees. The percentages after the PP variances show the ratio of PP values against the all-electron variances.

The nonlocal PP cutoff is generally lower for ccECPs than for eCEPPs (see Table 3). With the ccECPs and eCEPPs we got almost identical variances, which hints that the variance is stable against the cutoff radius.

**Table 3:** Nonlocal Cutoff Radius Values (Bohr) for Various Atoms, in Atomic Units, for eCEPPs and ccECPs^a^

atom	Be	B	C	N	O	F
eCEPP	2.735610	2.066929	1.603693	1.608516	1.427703	1.324644
ccECP	2.405539	1.897133	1.430486	1.341815	1.088303	1.039012

a The cutoff is defined as the radius above which the PP radial functions are less than 10^–6^ Ha.

Because the variance of the VMC energy is smaller with PPs, one can optimize the Jastrow factor with fewer Monte Carlo samples and hence less computational cost.

### Analysis of the Transcorrelated Integrals

For the systems studied in this work, we have investigated statistical parameters of the off-diagonal values of *V*, *K*, and *P* tensors, as well as the full TC Hamiltonian. The minima, maxima, mean values, and the Frobenius norms of the tensors are shown in Figure 2 for both ccECPs and eCEPPs. The values are shown in Hartrees. The Frobenius norm is defined as $F=\sqrt{\sum_{\mathrm{ij}}A_{\mathrm{ij}}^{2}}$ for a matrix *A*.

**Figure 2:**
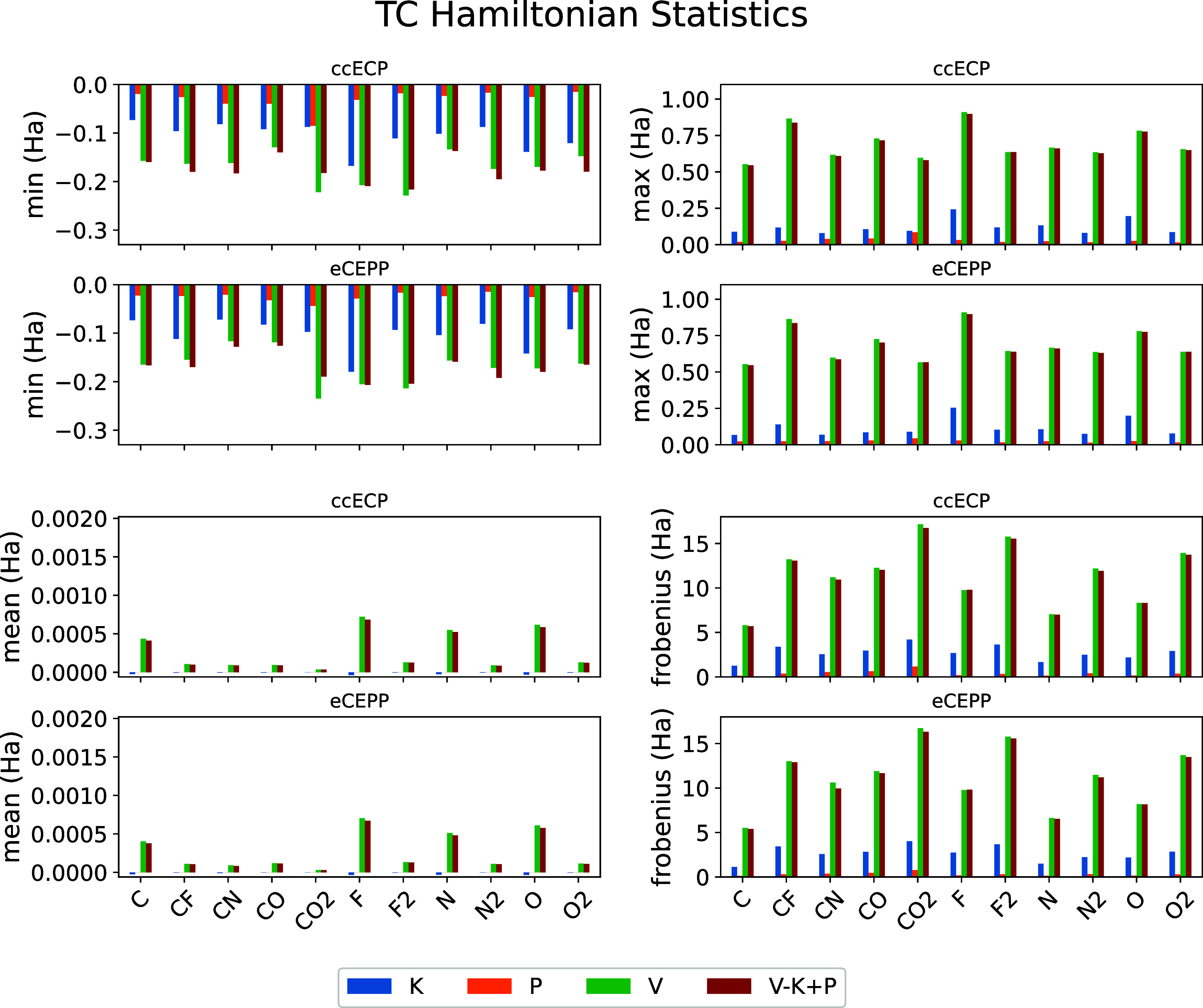
Parameters of the off-diagonal values of *V* (green), *K* (blue), *P* (orange), and *V* – *K* + *P* (red) tensors of eq 10 for different systems. The minima (upper left), maximas (upper right), mean values (lower left), and Frobenius norms (lower right) of the tensors are shown in Hartrees. For each stochastic parameter, we show values for each system for both ccECPs and eCEPPs.

The data shows that the largest values and overall weight of the off-diagonals are in the *V* tensor, i.e., the nontranscorrelated Hamiltonian, with the full TC Hamiltonian having slightly smaller values and overall weight (the Frobenius norm) than *V*. The *K* tensor of the kinetic energy operator commutators has much smaller weight compared to the full Hamiltonian, and the statistical parameters of the *P* tensor show that the pseudopotential commutators introduce only a slight correction to the TC Hamiltonian.

This data shows that pseudopotential commutators introduce generally small but non-negligible contributions.

### Atoms Be–F

#### Analysis of the Degree of PP Commutators

In Figure 3 we show the xTC–CCSD­(T)­(PP-*n*) total energies of the first row elements Be–F, along with their ionized states, as a function of *n*, the degree of PP commutators evaluated. Results with both eCEPPs (a) and ccECPs (b) are shown. The results are calculated with the quadruple-ζ basis set. The zero in the *y*-axis refers to *E*_CBS_^TZ–QZ^(CCSD­(T)) (see eq 14).

**Figure 3:**
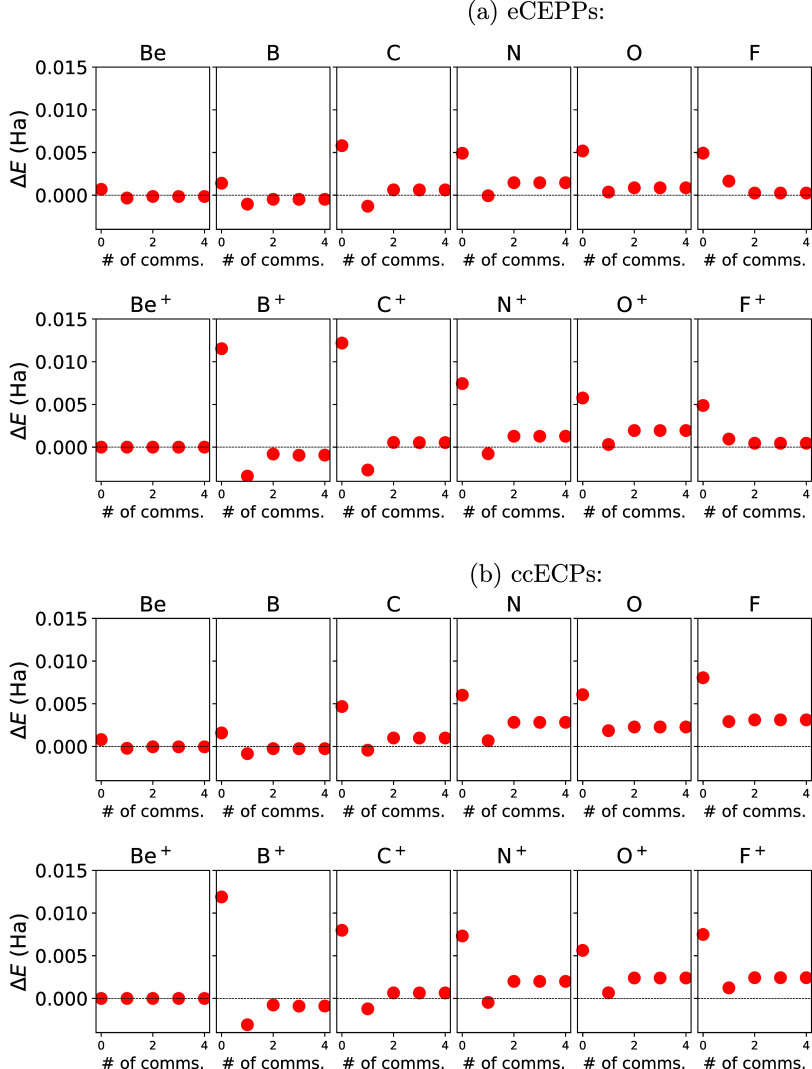
xTC–CCSD­(T)­(PP-*n*) energies with eCEPPs (figure (a), AVQZ basis) and ccECPs (figure (b), PVQZ basis) for the neutral and ionized states of the first row elements considered, shown as a function of the degree of PP commutators *n*. Results with *n* = 0, 1, 2, 3, 4 are shown. Results are presented relative to CBS estimate as Δ*E* = *E*(xTC–CCSD­(T) (PP–*n*)) – *E*_CBS_^TZ–QZ^(CCSD­(T)), see eq 14.

The Be cation with PPs has only one electron, and hence there is no correlation energy. With other atoms and ions, the general trend is that the xTC–CCSD­(T)­(PP-1) are smaller than the xTC–CCSD­(T)­(PP-0) energies, and the xTC–CCSD­(T)­(PP-2) energies increase slightly from the xTC–CCSD­(T)­(PP-1) energies. The energy converges with the second order commutator evaluation for all of the systems, except for the B ion, where the third order commutator evaluation still decreases the energy, although the difference is small.

Figure 4 shows the ionization energies of the first row elements Be–F, obtained with CCSD­(T), xTC–CCSD­(T)­(PP-0), xTC–CCSD­(T)­(PP-1), and xTC–CCSD­(T)­(PP-2) using both eCEPPs (a) and ccECPs (b). The results are shown as a function of the basis set. The ionization energies are shown against experimental values.^39^

**Figure 4:**
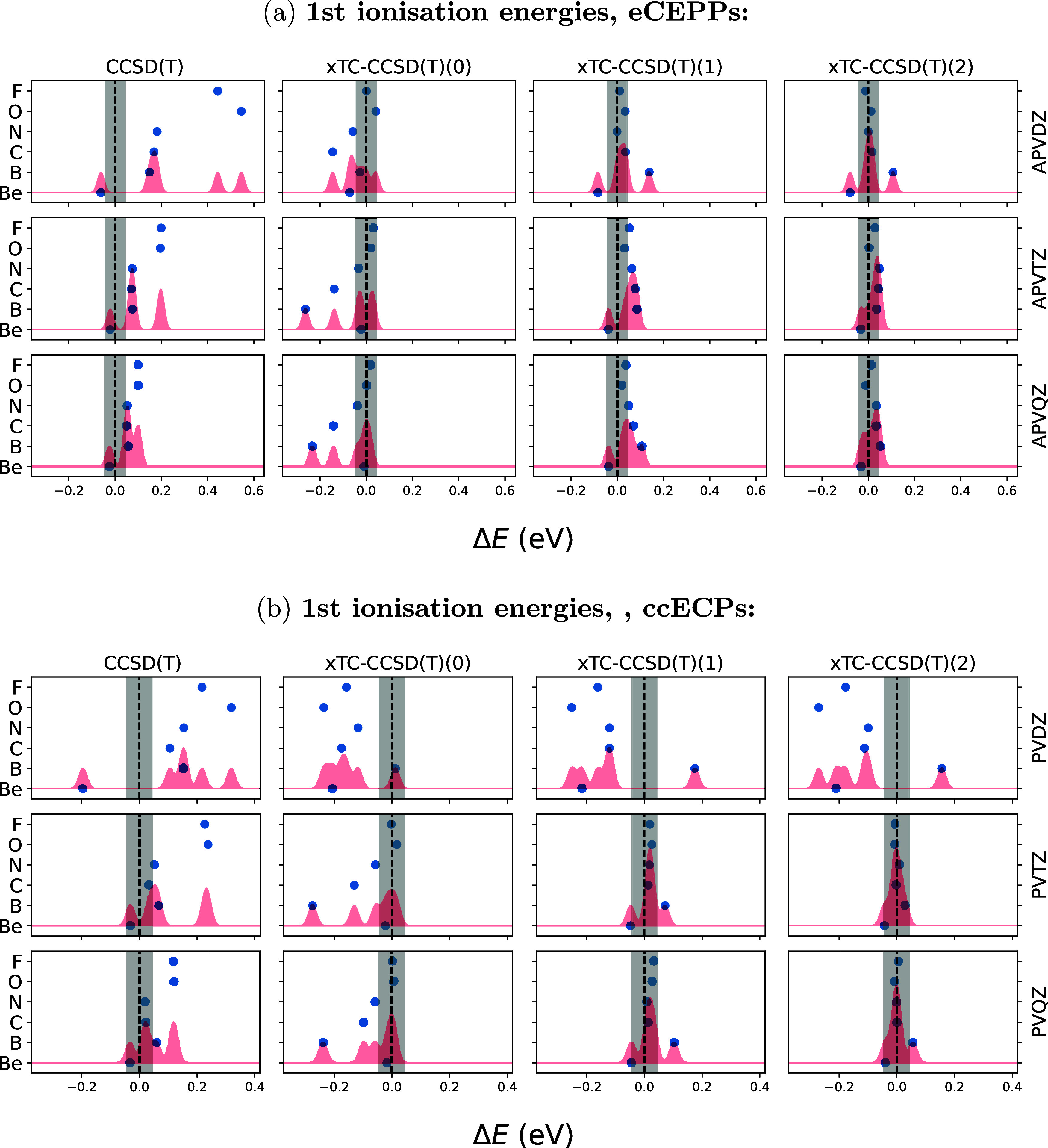
Ionization energies (IPs) *E*_*i*_ = *E*_atom_ – *E*_ion_ for the first row elements, using (a) eCEPPs and (b) ccECPs. The energies are presented as the discrepancy with the experimental ionization energies,^39^ so that the presented energies are Δ*E* = *E*_method_^basis^ – *E*_exp_. The *X*-axis shows the xTC–CCSD­(T)­(PP-*n*) ionization energies with *n* = 0, 1, 2 (in second, third, and fourth column, respectively). The first column shows CCSD­(T) ionization energies. The basis sets used are the AVXZ series for eCEPPs and the PVXZ series for ccECPs, where X is either D, T, or Q in the first, second, and third row, respectively. The gray shaded region denotes chemical accuracy. The red shading denotes a sum of gaussians, centered at each data point, with a width set such that equidistant gaussians in the presented scale would overlap at 95% confidence.

CCSD­(T) ionization energies reach chemical accuracy (1.6 mHa, 0.04 eV) for Be, C, and N with ccECPs and PVQZ basis set. With eCEPPs and AVQZ basis only Be ionization energy with CCSD­(T) is chemically accurate, but B, C, and N are close to chemical accuracy.

With xTC–CCSD­(T)­(PP-2) we reach chemical accuracy already with the AVTZ basis set for all of the atoms, with both PPs. The evaluation of the PP commutators is seen to be important, as with PP commutator degrees *n* < 2 the xTC–CCSD­(T)­(PP-*n*) ionization energies are generally worse.

Table 4 shows the mean absolute and root-mean-square errors (MAE and RMS) of the ionization energies, evaluated against experimental values, of the first row elements Be–F, obtained with CCSD­(T) and xTC–CCSD­(T)­(PP-*n*) methods and *n* = 0–4. The errors are averaged over the first-row atoms studied, and are shown separately for each basis set. Results are shown for both eCEPPs and ccECPs.

**Table 4:** Mean Absolute and Root Mean Square Errors (MAE and MSE) against the Experimental Ionization Energies of CCSD­(T) and xTC–CCSD­(T)­(PP-*n*) Methods with *n* = 0–4^a^

		eCEPP (eV)			ccECP (eV)		
# of comms.	error	AVDZ	AVTZ	AVQZ	PVDZ	PVTZ	PVQZ
CCSD(T)	MAE	0.2520	0.1001	0.0574	0.1846	0.1020	0.0560
	RMS	0.3047	0.1185	0.0624	0.1966	0.1330	0.0691
xTC–CCSD(T)(PP-0)	MAE	0.0630	0.0833	0.0737	0.1565	0.0899	0.0756
	RMS	0.0754	0.1234	0.1136	0.1724	0.1283	0.1099
xTC–CCSD(T)(PP-1)	MAE	0.0530	0.0520	0.0470	0.1803	0.0277	0.0338
	RMS	0.0694	0.0566	0.0568	0.1859	0.0371	0.0476
xTC–CCSD(T)(PP-2)	MAE	0.0435	0.0259	0.0266	0.1769	0.0191	0.0225
	RMS	0.0568	0.0303	0.0302	0.1858	0.0243	0.0296
xTC–CCSD(T)(PP-3)	MAE	0.0439	0.0267	0.0273	0.1772	0.0197	0.0231
	RMS	0.0574	0.0313	0.0314	0.1860	0.0251	0.0306
xTC–CCSD(T)(PP-4)	MAE	0.0439	0.0267	0.0272	0.1772	0.0197	0.0230
	RMS	0.0573	0.0312	0.0312	0.1860	0.0251	0.0306

a Results are obtained with eCEPPs and ccECPs across different basis sets. The errors are in eV.

Table 4 shows that CCSD­(T), xTC–CCSD­(T)­(PP-0), and xTC–CCSD­(T)­(PP-1) methods do not reach chemical accuracy with respect to MAE and RMS for the ionization energies of the first row elements with any of the basis sets. xTC–CCSD­(T)­(PP-0) is even worse in accuracy than CCSD­(T) in quadruple-ζ basis sets. However, xTC–CCSD­(T)­(PP-2) reaches chemical accuracy for both the MAE and MSE with both of the PPs at triple and quadruple-ζ basis sets.

It is interesting to note that xTC methods in double-ζ basis set are much better with eCEPPs than ccECPs, while the ccECPs are better with higher-order basis sets when *n* > 0. This phenomenon is not seen with standard CCSD­(T), where the accuracy is similar to both PPs at the same basis set cardinal number. Another feature visible in this table is that the best results with xTC–CCSD­(T) and ccECPs are obtained with the PVTZ basis set, and not with the PVQZ basis set, again when *n* > 0. This is not true with eCEPPs or with CCSD­(T). With eCEPPs and xTC–CCSD­(T)­(PP-2) the results are converged in AVTZ basis.

When comparing the results with 2 and 3 commutators evaluated, the MAE and MSE are within 1 mHa. The fourth commutator produces practically identical results to the third commutator.

To conclude this section, we have shown that it is necessary to include at least the second commutator, i.e., the PP-2 approximation, to achieve chemical accuracy in the ionization potentials of the first-row atoms with the xTC–CCSD­(T)-PP-*n* method. In other words, the first two nonzero commutators arising from the nonlocal pseudopotentials with the Jastrow factors are critically important to maintain reliability in the TC method with ECPs. Going to higher order commutators does not significantly change the results and hence can be disregarded. In further work we employ the PP-2 approximation.

### Molecules

#### Total Transcorrelated Energies

Figure 5 shows the energies of the molecules CN, CO, CF, N_2_, O_2_, F_2_, H_2_O, and CO_2_, obtained with eCEPPs (a) and ccECPs (b) with CCSD­(T) and xTC–CCSD­(T)­(PP-*n*) methods with *n* = 0 and *n* = 2. The energies are displayed relative to *E*_CBS_^TZ–QZ^(CCSD­(T)). The results are shown as a function of the basis set.

**Figure 5:**
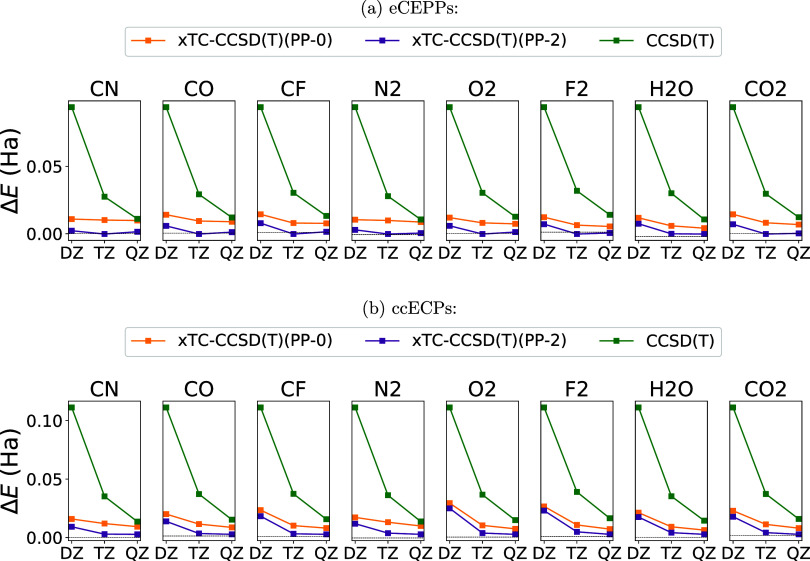
Energies of the molecules CN, CO, CF, N_2_, O_2_, F_2_, H_2_O, and CO_2_ with eCEPPs (a) and ccECPs (b) as a function of the basis set. The energies are relative to CCSD­(T) CBS energies as Δ*E* = *E* – *E*_CBS_^TZ–QZ^(CCSD­(T)), see eq 14. CCSD­(T) (green), xTC–CCSD­(T)­(PP-0) (yellow), and xTC–CCSD­(T)­(PP-2) (violet) energies are shown.

The xTC energies are always below CCSD­(T) energies at all basis sets. Evaluation of the PP commutators decreases the energies. Unlike with some atoms and ions, xTC–CCSD­(T)­(PP-0) is still lower in energy than CCSD­(T) for the molecules. The increase of the basis set size has a very small effect to the xTC–CCSD­(T) energies compared to the basis-set dependence of CCSD­(T) energies.

#### F12 Atomization Energies

Figure 6 shows the atomization energies of the molecules CN, CO, CF, N_2_, O_2_, F_2_, H_2_O, and CO_2_, calculated with CCSD­(T)-F12. The results are shown relative to the HEAT database.^27^ The results are obtained with the AVXZ basis sets, with X = D, T, Q. The results are shown for all-electron, eCEPP, and ccECP calculations.

**Figure 6:**
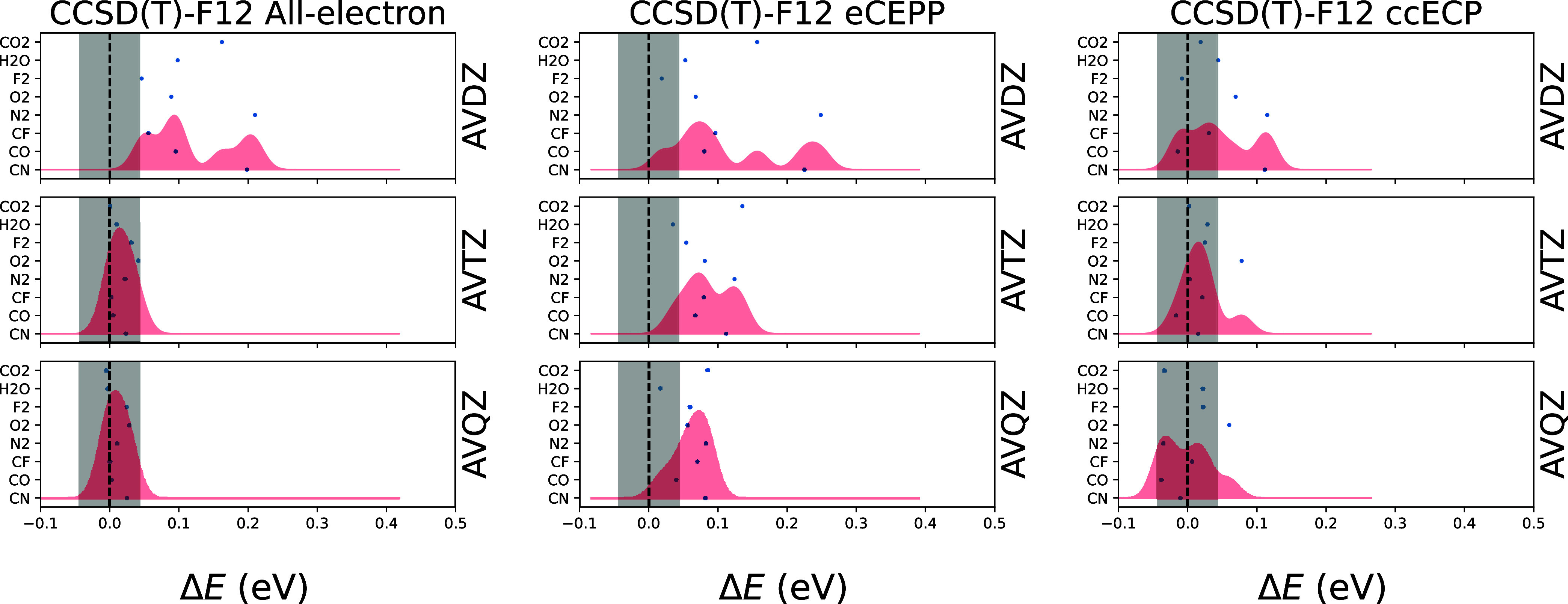
Atomization energies of the molecules CN, CO, CF, N_2_, O_2_, F_2_, H_2_O, and CO_2_, evaluated as *E*_at_ = ∑*E*_atom_ – *E*_mol_, calculated with CCSD­(T)-F12 method. The energies are presented relative to the results in the HEAT database, so that the presented energies are Δ*E* = *E*_HEAT_ – *E*_at_. Results are obtained using AVXZ basis sets, with X = D, T, Q (see labels on right). Results from all-electron (left), eCEPP (middle), and ccECP (right) calculations are shown. The gray shaded region denotes chemical accuracy. The red shading represents a sum of gaussians centered at the atomization energy discrepancies of each molecule, with sigmas set so that equidistantly placed gaussians over the interval between maximum and minimum of any calculation with a given core treatment (AE, eCEPP, ccECP) would have nearest-neighbor distance of 4σ.

The all-electron CCSD­(T)-F12 method shows excellent accuracy. With eCEPPs the accuracy is clearly worse, but then again with ccECPs all atoms are within chemical accuracy, except N_2_, which has a discrepancy of ∼50 meV.

Table 5 shows the MAE and RMS of the atomization energies of the molecules CN, CO, CF, N_2_, O_2_, F_2_, H_2_O, and CO_2_, obtained with CCSD­(T)-F12 and MRCI-F12 methods. The errors are averaged over the molecules studied, and are shown separately for each basis set and core treatment. CCSD­(T)-F12 is chemically accurate in AVTZ and AVQZ basis sets with all-electron calculations and with ccECPs both in terms of MAE and RMS.

**Table 5:** Mean Average and Root-Mean Square Errors (MAE and RMS) of the CCSD­(T)-F12 Atomization Energies of the Molecules Studied^a^

	all-electron			eCEPP			ccECP		
quantity	avdz	avtz	avqz	avdz	avtz	avqz	avdz	avtz	avqz
MAE	0.1133	0.0193	0.0135	0.1184	0.0861	0.0616	0.0515	0.0238	0.0283
RMS	0.1284	0.0235	0.0175	0.1417	0.0921	0.0656	0.0652	0.0326	0.0325

a The all-electron values, as well as ccECP and eCEPP pseudopotential results are shown.

The eCEPPs do not provide chemical accuracy. An interesting observation is that with eCEPPs the MAE and RMS decrease with increasing basis set size, but that the best MAE and RMS with ccECPs is obtained with the AVTZ basis set, and the AVQZ basis set is worse in MAE and almost equivalent in RMS. This decrease in accuracy with ccECPs when moving from triple to quadruple-ζ basis was already seen with the ionization energies of the first row elements and with the xTC–CCSD­(T) method.

**Table 6:** Mean Absolute Errors (MAE) and Root Mean Square Errors (RMS) for Different Methods

MAE						
	eCEPP			ccECP		
method	avdz	avtz	avqz	avdz	avtz	avqz
CCSD(T)	0.900	0.340	0.163	0.773	0.245	0.101
xTC–CCSD(T)(PP-0)	0.154	0.075	0.059	0.095	0.033	0.036
xTC–CCSD(T)(PP-2)	0.133	0.072	0.039	0.064	0.024	0.032
CCSDT	0.904	0.358	0.183	0.776	0.262	0.129
xTC–CCSDT(PP-0)	0.145	0.065	0.045	0.087	0.032	0.039
xTC–CCSDT(PP-2)	0.128	0.062	0.026	0.057	0.023	0.038

RMS						
	eCEPP			ccECP		
method	avdz	avtz	avqz	avdz	avtz	avqz
CCSD(T)	0.969	0.366	0.174	0.833	0.262	0.115
xTC–CCSD(T)(PP-0)	0.179	0.095	0.069	0.105	0.043	0.048
xTC–CCSD(T)(PP-2)	0.151	0.079	0.043	0.075	0.032	0.040
CCSDT	0.974	0.385	0.195	0.837	0.281	0.137
xTC–CCSDT(PP-0)	0.169	0.084	0.057	0.095	0.038	0.047
xTC–CCSDT(PP-2)	0.141	0.068	0.031	0.069	0.030	0.045

#### Atomization Energies with Transcorrelation

We calculated the atomization energies of the molecules CN, CO, CF, N_2_, O_2_, F_2_, H_2_O, and CO_2_, using eCEPPs and ccECPs with AVXZ basis sets (Figures 7 and 8). The results are presented as discrepancies relative to the HEAT values,^27^ employing CCSD­(T) and CCSDT.^30^ Both methods were applied in three variants: non-TC, xTC without PP commutator evaluations, and xTC with two PP commutator evaluations.

**Figure 7:**
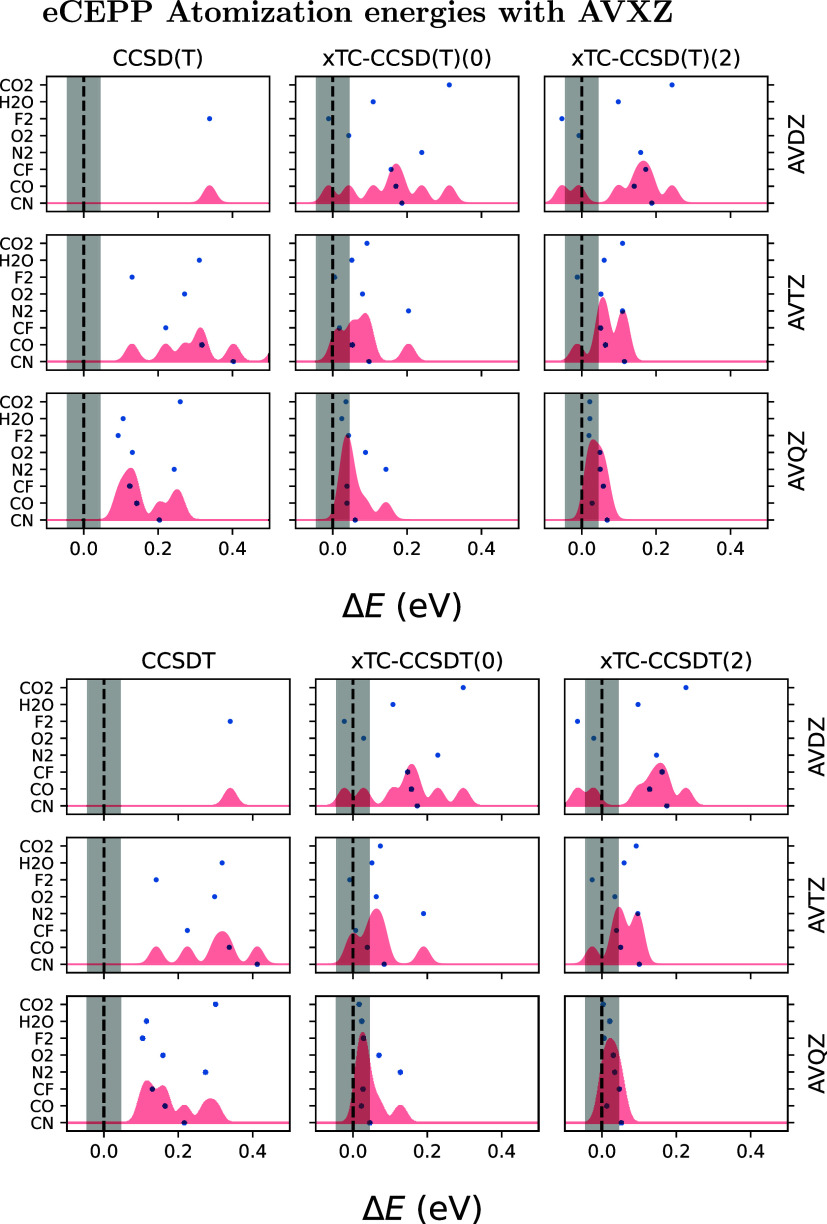
Atomization energies of a test set of molecules, evaluated with eCEPPs as *E*_at_ = ∑*E*_atom_ – *E*_mol_. The energies are presented relative to the results in the HEAT database, so that the presented energies are Δ*E* = *E*_HEAT_ – *E*_at_. Calculations are shown without TC (left column), and with xTC both without and with 2 PP commutator evaluations ({method}(0) and { method}(2), respectively, method being either CCSD­(T) or CCSDT). The gray shaded region denotes chemical accuracy. The red shading represents a sum of gaussians centered at the atomization energy discrepancies of each molecule, with sigmas set so that equidistantly placed gaussians over the interval between maximum and minimum of a given level of theory would have nearest-neighbor distance of 4σ. To improve presentation and to better compare with CCSD­(T)-F12 results, we are only showing the region −0.1 < Δ*E* < 0.5 eV, which leaves some of the energies of nontranscorrelated methods outside of the range in AVDZ and AVTZ basis sets.

**Figure 8:**
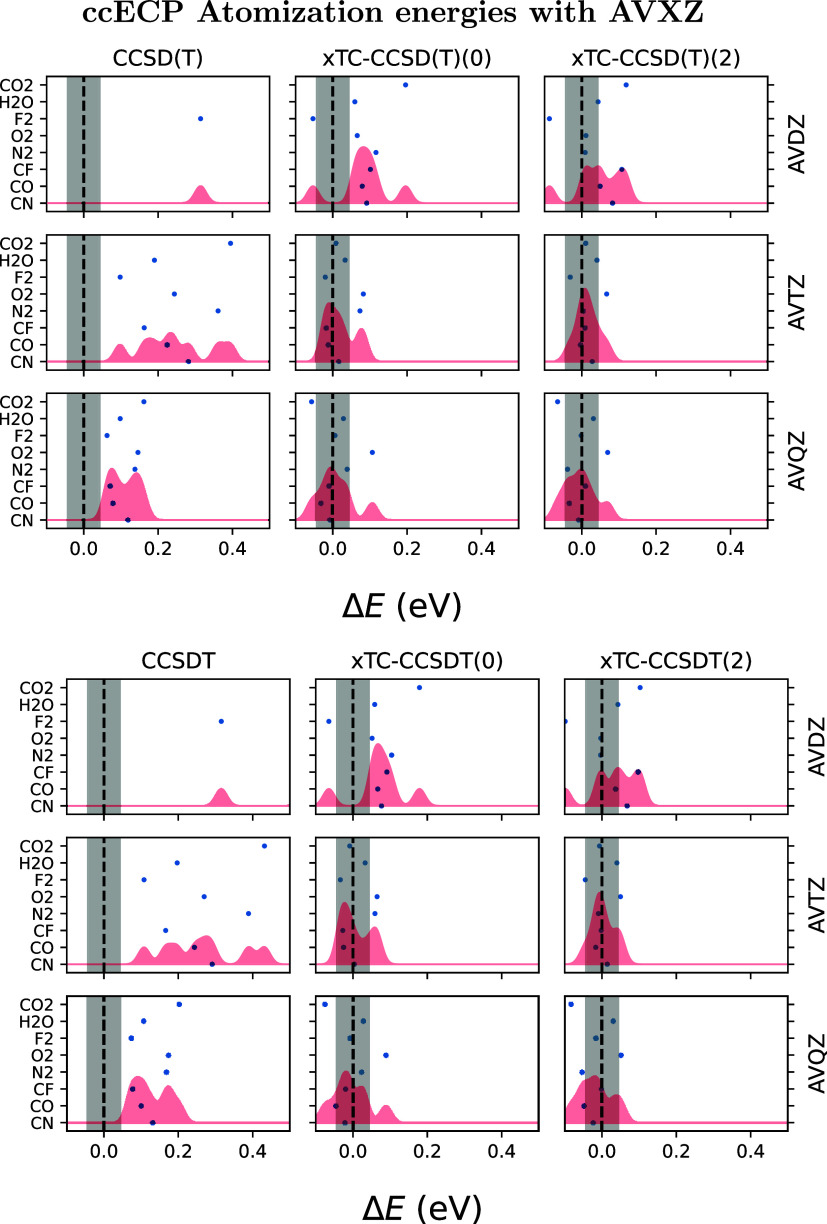
Results evaluated as in Figure 7, but with ccECPs and AVXZ (X = D, T, Q) basis sets.

The eCEPP results (Figure 7) show consistent improvement in accuracy with basis set size. Both xTC variants outperform the non-TC calculations with both CCSD­(T) and CCSDT, with PP commutator evaluations further enhancing accuracy. xTC–CCSDT(2) reaches chemical accuracy for all molecules studied. The results with ccECPs show best performance with xTC and ECP commutator evaluations in the AVTZ basis, where the results are even more accurate than with eCEPPs in AVQZ basis. However, the ccECP results in AVQZ basis, despite being within chemical accuracy (with CO_2_ slightly outside of the chemical accuracy regime), are slightly worsened from AVTZ results.

Table 6 summarizes the mean absolute errors (MAE) and root-mean-square errors (RMS) for all combinations of PP type, method, basis set, and theory. In terms of MAE and RMS, nontranscorrelated methods remain far from chemical accuracy even with the AVQZ basis set. In contrast, transcorrelated methods with eCEPPs, AVQZ basis sets, and two PP commutator evaluations achieve chemical accuracy across all studied methods. With ccECPs, xTC methods with two PP commutator evaluations are within or very close to chemical accuracy with the AVQZ basis, and within chemical accuracy using the AVTZ basis set. The fact that we see best results with the AVTZ basis, and not with the AVQZ basis, when using ccECPs both with F12 and transcorrelated methods hints that the nonmonotonic increase in accuracy with basis set resolution is a feature of the ccECPs.

### Dissociation Energies

#### N_2_

Figure 9 shows energy differences between experimental and theoretical evaluations of the N_2_ dissociation curve. Energy differences are presented as a function of bond length. The experimental curve is taken from ref 40. The theoretical curves are evaluated with FCIQMC and MRCI methods. We used eCEPPS and did the calculations in AVDZ, AVTZ, and AVQZ basis sets using FCIQMC. We show results for FCIQMC and xTC-FCIQMC­(PP-2). MRCI-F12 results are only evaluated in the AVQZ basis set. The theoretical results are shifted to overlap with experiment at *r* = 4.2 Å, a length which corresponds to the system of two isolated nitrogen atoms.

**Figure 9:**
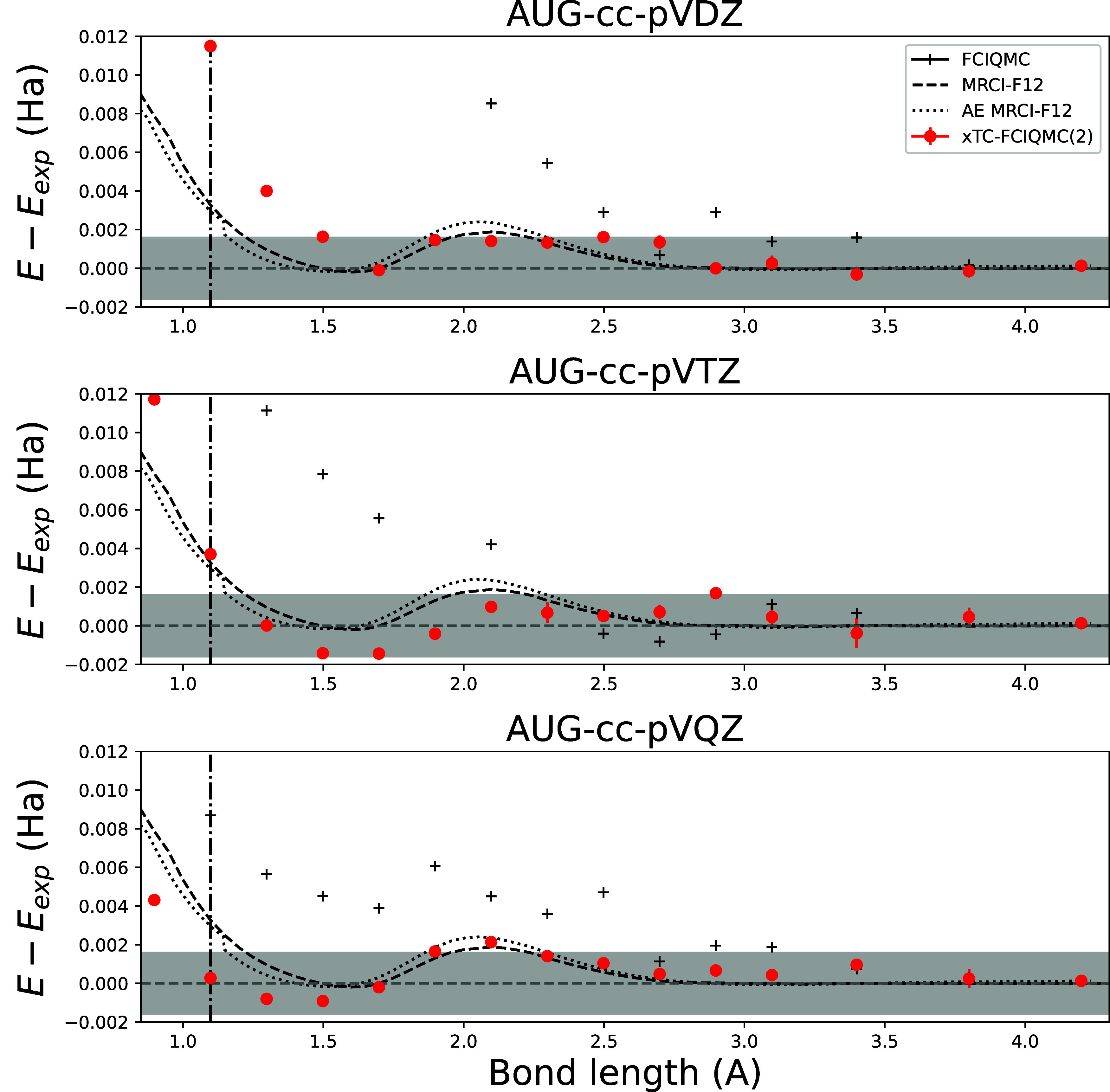
N_2_ FCIQMC (black crosses) and xTC-FCIQMC­(PP-2) (red circles) dissociation curves as a function of bond length with eCEPPS, computed in AVDZ (top row), AVTZ (middle row), and AVQZ (bottom row) basis sets. MRCI-F12 energies are evaluated in AVQZ basis set in all of the images, and both eCEPP (dashed black) and all-electron MRCI-F12 (dotted black) curves are shown. Results are presented as differences to the experimental dissociation curve from ref 40, taking the overlap between theory and experiment to be at *r* = 4.2 Å. The gray shaded region denotes chemical accuracy with respect to experiment. The dash-dotted vertical line points to the equilibrium bond length of N_2_ of *r* = 1.098 Å.

Because of the strong correlations and change of spin state involved in dissociation of the N_2_, the Hartree–Fock wave function is a poor reference for the Jastrow factor optimization. Hence we used a method developed recently to tackle this problem.^41^ First, we took the 100 most populated determinants from a nontranscorrelated FCIQMC calculation, and used them for constructing a trial wave function for VMC, and optimized the Jastrow factor with the VMC method before using it for preparing the transcorrelated Hamiltonian. In the subsequent xTC phase we used the reduced density matrix of the multideterminant wave function used in the optimization.

Figure 9 shows that the regular (nontranscorrelated) FCIQMC does not achieve chemical accuracy with respect to basis-set-error at near-equilibrium bond lengths. xTC-FCIQMC­(PP-2) underestimates the equilibrium energy at triple-ζ basis by about 4mH, but has an excellent match in quadruple-ζ-basis, a feature already seen with xTC coupled cluster methods and eCEPPs in Figure 7. The xTC-FCIQMC­(PP-2) method in quadruple-ζ-basis obtains chemical accuracy everywhere except at the most compressed bond length (below 1 Å), and at *r* = 2.098 Å, where, however, the stochastic error of the result overlaps with the chemical accuracy regime.

We tested the size-consistency by calculating the difference between the FCIQMC energies of two isolated nitrogen atoms and the energy of the N_2_ molecule at the longest bond distance studied, Δ*E* = 2*E*_N_ – *E*_N_2__ (*r* = 4.2 Å). This test was done in AVDZ basis, and resulted in Δ*E* = −1.5(2) mHa. This small size-inconsistency can be traced to the use of the combined Jastrow treatment (see discussion after eq 6) together with the xTC approximation, since the latter approximates the effect of the three-body interactions introduced by the commutators of the kinetic energy operator and the Jastrow factor. In the combined Jastrow approach the one-body terms are folded into 2-body Jastrow terms, which in turn leads to additional 3-body interactions (approximated in the xTC treatment). This combination leads to size-inconsistency. This size-consistency error can be entirely eliminated by using the separated Jastrow treatment (see Supporting Information for the separate treatment of PP commutators). We verified explicitly with further calculations that the xTC approximation, in the separate Jastrow treatment, does not incur a size-consistency error. Because we checked that in equilibrium geometries the separate and combined treatments yielded the same results, the overall error in this study due to the aforementioned size-inconsistency is on the order of 1–2 mHa. The best workflow for future studies will require further investigation of Jastrow cutoffs and the use of the separate Jastrow treatment.

The MRCI-F12 results are in good agreement with the experimental curve at longer distances, but overestimate the energy at equilibrium distance. The correspondence between the eCEPP and all-electron curves indicate that the eCEPPs introduce almost no error into the simulation.

#### F_2_

Figure 10 shows the dissociation error curve of F_2_, showing the difference between MRCI-F12 and xTC-FCIQMC­(PP-2) theoretical methods and experiment. xTC-FCIQMC­(PP-2) results are evaluated with eCEPPS and computed in AVDZ, AVTZ, and AVQZ basis sets. The MRCI-F12 results are evaluated in the AVQZ basis set. The experimental curve is taken from ref 42. Because the experimental data extends only to *r* ∼ 2.8 Å, the theoretical results are shifted to overlap with experiment at the equilibrium bond length of *r* = 1.4118 Å.

**Figure 10:**
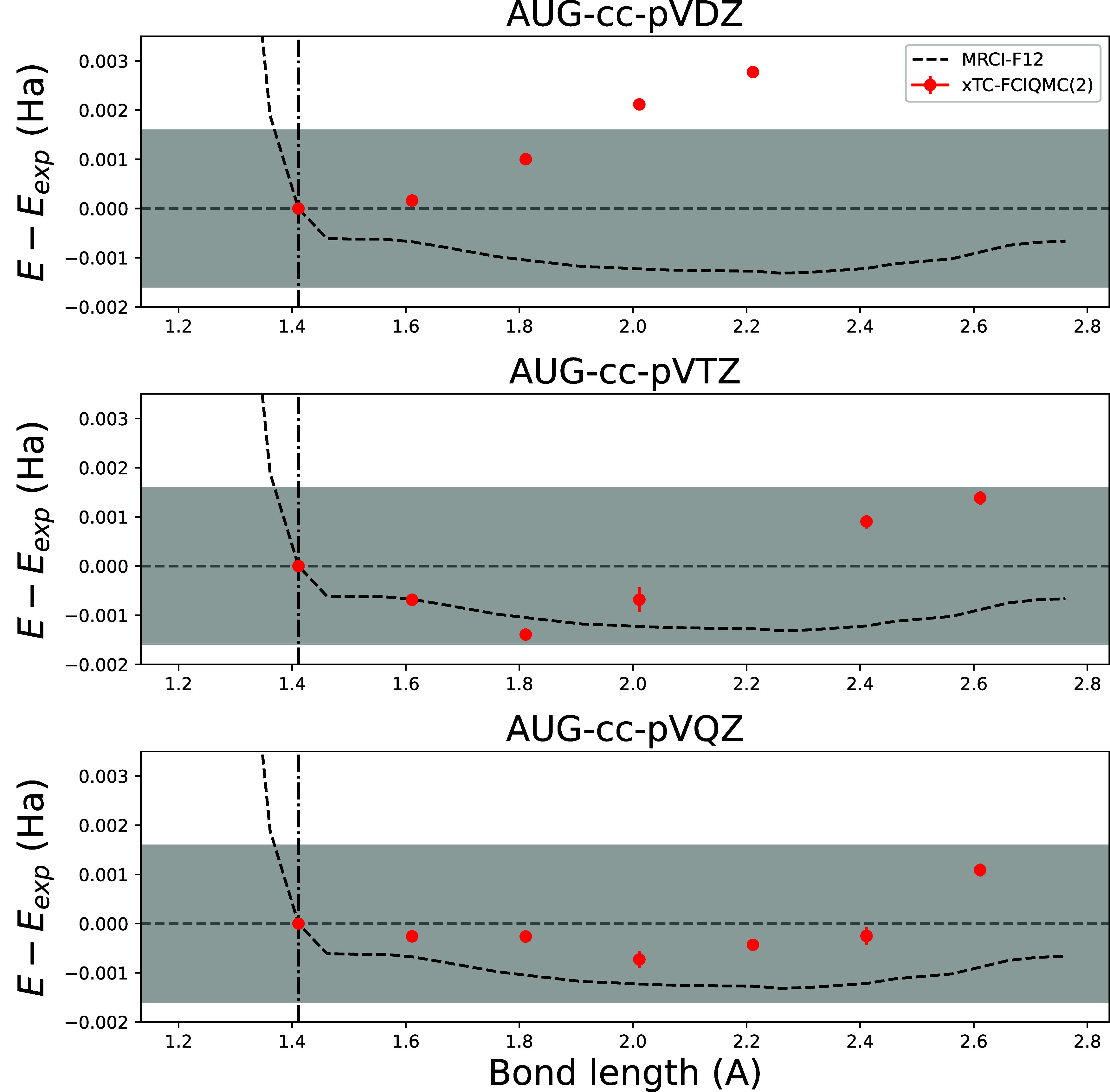
F_2_ xTC-FCIQMC­(PP-2) and MRCI-F12 energies as a function of bond length with eCEPPS, computed in AVDZ (top row), AVTZ (middle row) and AVQZ (bottom row) basis sets. MRCI-F12 energies are the same in all plots, and are evaluated in AVQZ basis set. Results are presented as differences to experimental dissociation curve from ref 42. The gray shaded region denotes chemical accuracy with respect to experiment. The dotted vertical line points to the equilibrium bond length of F_2_ of 1.4112 Å.

The Jastrow factors for F_2_ are optimized with only the RHF determinant in the VMC method. This proves to be already highly accurate, as for F_2_ only a single bond is broken and less correlation is involved in the dissociation.

The xTC-FCIQMC­(PP-2) results at triple-ζ and higher basis sets are very accurate, with a sub-mHa error along the whole range of the plot with AVQZ basis set. MRCI-F12 is also chemically accurate with the AVQZ basis set, but the error is larger.

## Conclusions

We have presented a study of transcorrelated theory under PP approximations. The study included the derivation of the PP commutators needed for evaluating the transcorrelated Hamiltonian. The algorithms were implemented using an in-house code TCHINT, that was subsequently used to evaluate the accuracy of the transcorrelated methods with PPs.

The accuracy of the method was evaluated by estimating the ionization potentials of atoms in the first row, the atomization energies of a test set of molecules, and the dissociation curves of N_2_ and F_2_. The results were compared to the HEAT database, experimental data, and MRCI-F12 results.

xTC–CCSD­(T)­(PP-2) provided chemical accuracy for the ionization energies of the first row atoms with both eCEPPs and ccECPs. For the atomization energies, all of the coupled cluster levels of theory with 2 PP commutator evaluations reached chemical accuracy with eCEPPs and AVQZ basis set. With ccECPs, chemical accuracy was reached with AVTZ basis set, while with AVQZ basis the RMS of the methods was slightly outside the chemical accuracy regime.

Atomization energy calculations with CCSD­(T)-F12 showed the same trend of reduced accuracy when moving from AVTZ to AVQZ basis. This leads to the conclusion that with explicitly correlated methods, the ccECPs have some problems with augmented basis set convergence, but that the results are very accurate already at triple-ζ level. The F12 methods were found to work better with ccECPs than eCEPPs.

Finally, the dissociation curves of N_2_ and F_2_ were evaluated with xTC-FCIQMC and MRCI-F12 methods. The xTC-FCIQMC results were very accurate with the AVQZ basis set and, apart from the compressed distance region of N_2_, chemical accuracy was reached.

The results of this study show that the transcorrelated methods are very accurate with PPs, and that the accuracy is comparable to the all-electron results. The evaluation of the PP commutators is essential for the accuracy of the method. It is possible that optimization of the Jastrow factor without the presence of the core electrons allows a more targeted TC simulation, focusing on the valence electrons, a feature than can prove useful in the future development of the method.

The theory of transcorrelation with PPs can help bring the applicability of the TC method to a wider range of systems. Calculations with larger system sizes can benefit from the reduced variance, making the Jastrow optimizations more feasible. Applications with heavier atoms, such as transition metals, would be an interesting future direction. Crucially, the methods presented in this work can help in the development of TC theory toward periodic solid-state systems, possibly even in the plane-wave basis. Directions toward application of TC theory with PPs in embedding models for systems such as solid-state defects are also currently under investigation.

## Supplementary Material





## Notes

Optimized Jastrow factor parameters for atoms and molecules (ZIP)
Derivations of pseudopotential commutator formulas (separate Jastrow treatment), and
FCIDUMP files containing transcorrelated second-quantized Hamiltonians for Be in AVDZ basis set, with and without the PP commutator evaluations (PDF)

## References

[1] Kato T. (1957). On the eigenfunctions
of many-particle systems in quantum mechanics. Commun. Pure Appl. Math..

[2] Kutzelnigg W. (1985). r 12-Dependent
terms in the wave function as closed sums of partial wave amplitudes
for large l. Theor. Chim. Acta.

[3] Ten-no S. (2004). Initiation
of explicitly correlated Slater-type geminal theory. Chem. Phys. Lett..

[4] Kutzelnigg W., Klopper W. (1991). Wave functions with terms linear
in the interelectronic
coordinates to take care of the correlation cusp. I. General theory. J. Chem. Phys..

[5] Noga J., Kutzelnigg W. (1994). Coupled cluster theory that takes care of the correlation
cusp by inclusion of linear terms in the interelectronic coordinates. J. Chem. Phys..

[6] Kong L., Bischoff F. A., Valeev E. F. (2012). Explicitly Correlated R12/F12 Methods
for Electronic Structure. Chem. Rev..

[7] Cohen A. J., Luo H., Guther K., Dobrautz W., Tew D. P., Alavi A. (2019). Similarity
transformation of the electronic Schrödinger equation via Jastrow
factorization. J. Chem. Phys..

[8] Ammar A., Scemama A., Giner E. (2023). Transcorrelated selected configuration
interaction in a bi-orthonormal basis and with a cheap three-body
correlation factor. J. Chem. Phys..

[9] Ammar A., Scemama A., Giner E. (2023). Biorthonormal Orbital Optimization
with a Cheap Core-Electron-Free Three-Body Correlation Factor for
Quantum Monte Carlo and Transcorrelation. J.
Chem. Theory Comput..

[10] Lee N., Thom A. J. W. (2023). Studies on the Transcorrelated Method. J. Chem. Theory Comput..

[11] Ten-no S. L. (2023). Nonunitary
projective transcorrelation theory inspired by the F12 ansatz. J. Chem. Phys..

[12] Ammar A., Scemama A., Loos P.-F., Giner E. (2024). Compactification of
determinant expansions via transcorrelation. J. Chem. Phys..

[13] Haupt J. P., Hosseini S. M., Ríos P. L., Dobrautz W., Cohen A., Alavi A. (2023). Optimizing Jastrow
factors for the transcorrelated method. J. Chem.
Phys..

[14] Dobrautz W., Luo H., Alavi A. (2019). Compact numerical solutions to the two-dimensional
repulsive Hubbard model obtained via nonunitary similarity transformations. Phys. Rev. B.

[15] Christlmaier E. M. C., Schraivogel T., Ríos P. L., Alavi A., Kats D. (2023). xTC: An efficient
treatment of three-body interactions in transcorrelated methods. J. Chem. Phys..

[16] Luo H., Alavi A. (2018). Combining the Transcorrelated
Method with Full Configuration Interaction
Quantum Monte Carlo: Application to the Homogeneous Electron Gas. J. Chem. Theory Comput..

[17] Liao K., Schraivogel T., Luo H., Kats D., Alavi A. (2021). Towards efficient
and accurate ab initio solutions to periodic systems via transcorrelation
and coupled cluster theory. Phys. Rev. Res..

[18] Guther K., Cohen A. J., Luo H., Alavi A. (2021). Binding curve of the
beryllium dimer using similarity-transformed FCIQMC: Spectroscopic
accuracy with triple-zeta basis sets. J. Chem.
Phys..

[19] Schraivogel T., Cohen A. J., Alavi A., Kats D. (2021). Transcorrelated coupled
cluster methods. J. Chem. Phys..

[20] Schraivogel T., Christlmaier E. M. C., Ríos P. L., Alavi A., Kats D. (2023). Transcorrelated
coupled cluster methods. II. Molecular systems. J. Chem. Phys..

[21] Sokolov I. O., Dobrautz W., Luo H., Alavi A., Tavernelli I. (2023). Orders of
magnitude increased accuracy for quantum many-body problems on quantum
computers via an exact transcorrelated method. Phys. Rev. Res..

[22] Trail J. R., Needs R. J. (2017). Shape and energy
consistent pseudopotentials for correlated
electron systems. J. Chem. Phys..

[23] Bennett M. C., Melton C. A., Annaberdiyev A., Wang G., Shulenburger L., Mitas L. (2017). A new generation of effective core potentials for correlated calculations. J. Chem. Phys..

[24] Fahy S., Wang X. W., Louie S. G. (1990). Variational
quantum Monte Carlo nonlocal
pseudopotential approach to solids: Formulation and application to
diamond, graphite, and silicon. Phys. Rev. B.

[25] Drummond N. D., Towler M. D., Needs R. J. (2004). Jastrow
correlation factor for atoms,
molecules, and solids. Phys. Rev. B.

[26] TCHINT, To be published.

[27] Tajti A., Szalay P. G., Császár A. G., Kállay M., Gauss J., Valeev E. F., Flowers B. A., Vázquez J., Stanton J. F. (2004). HEAT: High accuracy extrapolated
ab initio thermochemistry. J. Chem. Phys..

[28] Sun Q., Zhang X., Banerjee S. (2020). Recent developments
in the PySCF program package. J. Chem. Phys..

[29] Needs R. J., Towler M. D., Drummond N. D., Ríos P. L., Trail J. R. (2020). Variational and diffusion quantum Monte Carlo calculations
with the CASINO code. J. Chem. Phys..

[30] Kats, D. ; Schraivogel, T. ; Hauskrecht, J. ; Rickert, C. ; Simula, K. ; Wu, F. ElemCo.jl,: Julia program package for electron correlation methods. 2024.

[31] Kats D., Christlmaier E. M., Schraivogel T., Alavi A. (2024). Orbital optimization
in xTC transcorrelated methods. Faraday Discuss..

[32] Werner H.-J., Knowles P. J., Knizia G., Manby F. R., Schütz M. (2012). Molpro: a
general-purpose quantum chemistry program package. WIREs Comput. Mol. Sci..

[33] Werner H.-J., Knowles P. J., Manby F. R., Black J. A., Doll K., Heß A., elmann A., Kats D., hn Kö A., Korona T., Kreplin D. A. (2020). The Molpro quantum chemistry
package. J. Chem. Phys..

[34] Werner, H.-J. ; Knowles, P. J. MOLPRO, a package of ab initio programs. https://www.molpro.net/.

[35] Peterson K. A., Adler T. B., Werner H.-J. (2008). Systematically
convergent basis sets
for explicitly correlated wavefunctions: The atoms H, He, B-Ne, and
Al-Ar. J. Chem. Phys..

[36] Cleland D., Booth G. H., Alavi A. (2010). Communications: Survival of the fittest:
Accelerating convergence in full configuration-interaction quantum
Monte Carlo. J. Chem. Phys..

[37] Guther K., Anderson R. J., Blunt N. S. (2020). NECI: N-Electron Configuration
Interaction with an emphasis on state-of-the-art stochastic methods. J. Chem. Phys..

[38] Davidson E. R., Silver D. W. (1977). Size consistency
in the dilute helium gas electronic
structure. Chem. Phys. Lett..

[39] Chakravorty S. J., Gwaltney S. R., Davidson E. R., Parpia F. A., Parpia F. A., Fischer C. F. p. (1993). Ground-state
correlation energies for atomic ions with
3 to 18 electrons. Phys. Rev. A.

[40] Le
Roy R. J., Huang Y., Jary C. (2006). An accurate analytic
potential function for ground-state N_2_, from a direct-potential-fit
analysis of spectroscopic data. J. Chem. Phys..

[41] Haupt, J. P. To be published.

[42] Colbourn E. A., Dagenais M., Douglas A. E., Raymonda J. W. (1976). The electronic spectrum
of F2. Can. J. Phys..

