# Supplementary material for: Transcorrelated Theory with Pseudopotentials
Source: J Chem Theory Comput. 2025 May 13;21(10):5155–70. doi: 10.1021/acs.jctc.5c00343 (PMC12120921; doi:10.1021/acs.jctc.5c00343)
Supplement: Supplementary file 2 [file ct5c00343_si_002.pdf]

Transcorrelated Theory with Pseudopotentials: Supplemental Information

Kristoffer Simula,[*,†] Evelin Martine Corvid Christlmaier,[†] Maria-Andreea Filip,[†] J. Philip Haupt,[†] Daniel Kats,[†] Pablo Lopez-Rios,[†] and Ali Alavi[*,†,‡]

†*Max Planck Institute for Solid State Research, Heisenbergstr. 1, 70569 Stuttgart, Germany*

‡*Yusuf Hamied Department of Chemistry, University of Cambridge, Lensfield Road, Cambridge CB2 1EW, United Kingdom*

E-mail: k.simula@fkf.mpg.de; a.alavi@fkf.mpg.de

If one wants to separate the treatment of the 1- and 2-body terms in the Jastrow factor when calculating the pseudopotential commutators, then the TC commutators arising from the 1-body Jastrow factor for 1-body operators $\hat{O}$,

$$P_q^p = \left\langle \phi_p \left| \Pi^1(\hat{O}) + \Pi^2(\hat{O}) + \Gamma^1(\hat{O}) + \Gamma^2(\hat{O}) \right| \phi_q \right\rangle \tag{1}$$

would be included in the 1-body matrix elements $< p|q >$ of the Hamiltonian, with

$$\begin{aligned} \Pi^i(\hat{H}_{en}^{PP}) &= \hat{H}_{en}^{PP}(\mathbf{r}_i)\sum_I^M \chi(\mathbf{r}_{iI}) - \sum_I^M \chi(\mathbf{r}_{iI})\hat{H}_{en}^{PP}(\mathbf{r}_i) \\ \Gamma^i &= \hat{H}_{en}^{PP}(\mathbf{r}_i)\left[\sum_I^M \chi(\mathbf{r}_{iI})\right]^2 + \left[\sum_I^M \chi(\mathbf{r}_{iI})\right]^2 \hat{H}_{en}^{PP}(\mathbf{r}_i) - 2\sum_I^M \chi(\mathbf{r}_{iI})\hat{H}_{en}^{PP}(\mathbf{r}_i)\sum_J^M \chi(\mathbf{r}_{iJ}). \end{aligned} \tag{2}$$

The 2-body commutators arising from the 2-body Jastrow factor for 1-body operators $\hat{O}$,

$$P_{rs}^{pq} = \left\langle \phi_p \phi_q \left| \Pi_{12}^1(\hat{H}_{en}^{PP}) + \Pi_{12}^2(\hat{H}_{en}^{PP}) + \frac{1}{2}\Gamma_{1212}^1(\hat{H}_{en}^{PP}) + \frac{1}{2}\Gamma_{1212}^2(\hat{H}_{en}^{PP}) \right| \phi_r \phi_s \right\rangle \tag{3}$$

are calculated with

$$\begin{aligned}
\Pi_{ij}^i(\hat{H}_{en}^{PP}) =& + \hat{H}_{en}^{PP}(\mathbf{r}_i)\theta(\mathbf{r}_i,\mathbf{r}_j) - \theta(\mathbf{r}_i,\mathbf{r}_j)\hat{H}_{en}^{PP}(\mathbf{r}_i), \\
\Gamma_{ijij}^i(\hat{H}_{en}^{PP}) =& \hat{H}_{en}^{PP}(\mathbf{R}_i)\theta(\mathbf{r}_i,\mathbf{r}_j)^2 + \theta(\mathbf{r}_i,\mathbf{r}_j)^2\hat{H}_{en}^{PP}(\mathbf{r}_i) \\
& -2\theta(\mathbf{r}_i,\mathbf{r}_j)\hat{H}_{en}^{PP}(\mathbf{r}_i)\theta(\mathbf{r}_i,\mathbf{r}_j) \\
& +2\chi(\mathbf{r}_i)\left(\theta(\mathbf{r}_i,\mathbf{r}_j)\hat{H}_{en}^{PP}(\mathbf{r}_i) - \hat{H}_{en}^{PP}(\mathbf{r}_i)\theta(\mathbf{r}_i,\mathbf{r}_j)\right) \\
& +2\left(\hat{H}_{en}^{PP}(\mathbf{r}_i)\theta(\mathbf{r}_i,\mathbf{r}_j) - \theta(\mathbf{r}_i,\mathbf{r}_j)\hat{H}_{en}^{PP}(\mathbf{r}_i)\right)\chi(\mathbf{r}_i).
\end{aligned} \tag{4}$$

with $\theta(\mathbf{r}_i,\mathbf{r}_j) = \left[u(\mathbf{r}_i,\mathbf{r}_j) + \sum_I^M f(\mathbf{r}_i,\mathbf{r}_j,\mathbf{R}_I)\right]$.
